# Effects of semi-deep water irrigation on hybrid indica rice lodging resistance

**DOI:** 10.3389/fpls.2022.1038129

**Published:** 2022-12-15

**Authors:** Hui Gao, Zhi Dou, Linrong Chen, Yao Lu, Dong Sun, Qiang Xu, Ruyin Sun, Xueying Chen

**Affiliations:** ^1^ Jiangsu Co-Innovation Center for Modern Production Technology of Grain Crops, Yangzhou University, Yangzhou, China; ^2^ Jiangsu Provincial Key Laboratory of Crop Genetics and Physiology, Yangzhou University, Yangzhou, China; ^3^ Research Institute of Rice Industrial Engineering Technology, Yangzhou University, Yangzhou, China; ^4^ Tianchang Modern Agricultural Industrial Park Management Service Center, Chuzhou, China; ^5^ Tianchang Agriculture Science and Technology Extension Centre, Chuzhou, China

**Keywords:** hybrid indica rice, semi-deep water irrigation, lodging resistance, mechanics, structural carbohydrate, anatomy

## Abstract

Recently, rice-aquatic animal integrated farming (RAAIF) has grown rapidly in China due to its favorable benefits and the lower application of pesticides and fertilizers. However, rice lodging occurs frequently under RAAIF, which restricts rice yield. We assumed that semi-deep water irrigation may cause weaker rice-lodging resistance since it is the most significant environmental factor for RAAIF that distinguishes it from rice monoculture. To investigate the response of rice stem lodging resistance to semi-deep water irrigation and its mechanism, three irrigation management modes, namely the typical high-yield irrigation model that is mainly based on swallow and wetting (CK), semi-deep water irrigation from the late tillering stage to the jointing stage (SDI1), and semi-deep water irrigation from the jointing stage to the middle grain-filling stage (SDI2), were conducted using three hybrid indica rice varieties: Shenliangyou136 (SLY136), Huiliangyousimiao (HLYSM), and Wanxiangyou982 (WXY982). Mechanics analysis indicated that the bending moment by the whole plant (WP) and the breaking strength (M) were both decreased by semi-deep water irrigation when compared with CK, while M presented a larger decreasing amplitude than WP, which induced the increased lodging index (LI) of rice, for all the tested varieties. SLY136 and HLYSM were affected more strongly by SDI1, whereas WXY982 was affected more strongly by SDI2. Significant weaker breaking force under two semi-deep water irrigation modes contributed to the decreased M relative to CK. Morphology results showed that semi-deep water irrigation reduced the thickness of mechanical tissues, sclerenchyma cells, and parenchyma cells; reduced the number of vascular bundles; and caused a looser arrangement, inducing the lower fullness of the rice basal internode. Decreased accumulation of lignin and cellulose was also linked to the weaker breaking force of the basal internode under semi-deep water irrigation, which was verified by correlation analysis. WXY982 had obvious lower structural carbohydrates content under semi-deep water irrigation than the other two varieties and thus showed worse breaking force and LI. In conclusion, the worse mechanical strength of the rice basal internode under semi-deep water irrigation was closely associated with weaker vascular bundle development and suppressed structural carbohydrate accumulation, and the decreasing degree of lodging resistance varied between rice varieties and semi-deep water irrigation periods.

## 1 Introduction

Rice-aquatic animal integrated farming (RAAIF) is an integrated system with rice cultivation and aquatic animal culture in the same paddy field based on its wetland resource, thereby acquiring products of both rice and aquatic animals with favorable economic and ecological benefits (Frei and Becker, 2005; Xie et al., 2011; Hou et al., 2021). In recent years, some RAAIF modes have expanded rapidly in China, especially in the south, with abundant freshwater resources such as rice–crayfish, rice–carp, and rice–soft shell turtle. By 2020, China’s RAAIF area had increased to 2.23 million, accounting for 7.8% of the total rice planting area (National Fisheries Technology Extension Center, 2021).

Semi-deep water irrigation is the most significant environmental factor for RAAIF that distinguishes it from rice monoculture. To satisfy the activity and growth of aquatic animals, farmers would carry out semi-deep water irrigation proactively during the given rice growth period in paddy fields, which usually reach 20- to 50-cm water depth (Liu J. et al., 2018). The irrigation scheme of RAAIF not only differs from high-yielding rice irrigation that is mainly based on swallow and wetting (Yao et al., 2012; Ye et al., 2013) but also fails to reach the water depth of deep-water rice, which is usually cultivated with more than 50-cm water depth (Kende et al., 1998); thus, we defined it as semi-deep water irrigation in the present study. In practice, when to conduct semi-deep water irrigation mainly depends on the local climate condition and requirement from specific aquatic animal culture.

Our research team found that rice lodging occurred more frequently in RAAIF than rice monoculture according to a previous survey, which had turned into a key limiting factor for realizing high rice yield. We speculated that higher lodging risk may be primarily associated with long-term semi-deep water irrigation. Rice lodging was divided into root lodging and stem lodging according to the lodging position, and the former was almost observed under a direct seeding and throwing seedling pattern, which is rarely found under the transplanting pattern. In RAAIF production, farmers mostly use the transplanting pattern to plant rice and prefer manually transplanting with long-age seedling, with the aim of releasing animal germchit and control weed by enough water depth after transplanting. Evidently, the lodging types under RAAIF were almost all stem lodging in accordance with our survey. Rice stem lodging resistance compactly correlated with the plant type, internode configuration, and the mechanical strength of basal internodes (Shah et al., 2019). Previous studies had indicated that an optimal balance between rice high yield and lodging resistance can be synchronously realized by properly shortening basal internodes, increasing the internode length below the panicle, and improving the plumpness (Zhang et al., 2014; Zheng et al., 2017; Liu Q. et al., 2018). The rice mechanical strength of basal internodes had positive relationship with mechanical tissue thickness and vascular bundle area and density, as claimed by many previous studies (Ookawa et al., 2014; Wu et al., 2017; Gui et al., 2018). It had been widely certified that the improved accumulation and proportion of structural carbohydrate including lignin and cellulose were conducive to enhancing the mechanical strength of the rice basal internode and lowering its lodging risk (Ookawa et al., 2014; Zhang et al., 2016; Mulsanti et al., 2018).

By far, many studies have examined the role of irrigation management in regulating rice growth and development, while most focused on rice yield and physical performance under short-term flooding and even submergence (Chu et al., 2017; Zhu et al., 2019), or the regulation effect of water-saving irrigation methods on rice leaf photosynthesis, grain filling, water use efficiency, and greenhouse gas emission (Belder et al., 2005; Ohe et al., 2010; Wang et al., 2020). Up to now, the influence of semi-deep water irrigation on rice development had been rarely reported, particularly the response of lodging resistance and related mechanism.

Compared with rice monoculture, RAAIF not only alters the depth of irrigation water during the specific rice growth phase but also brings several possible variable factors into the rice growth environment, such as the behavior of cocultured animals, fodder application, and waterweed returning. However, expounding the effects induced by semi-deep water irrigation was no doubt the foundation of further understanding of the multiple effects of an integrated farming system on rice growth. The purpose of this study was to clarify the influence of semi-deep water irrigation on indica rice lodging resistance and its related mechanics, morphology, and physiology mechanism; the response difference between genotypes was also analyzed. The results can help in elucidating the response of rice lodging resistance to semi-deep water irrigation and provide reference for rice cultivation with favorable lodging resistance under RAAIF.

## 2 Materials and methods

### 2.1 Site description

This experiment was carried out at the RAAIF experimental station of Yangzhou University in 2020. The site is located in Yongfeng County, Tianchang City, Anhui Province (32°72’94’’ N, 118°99’73’’ E), which is a typical RAAIF production district in China. The meteorological data are presented in **Supplementary Figure 1**. The properties of soil are as follows: alluvial loam, pH 6.0, total N 1.83 g kg^-1^, available phosphorus 17.8 mg kg^-1^, and rapidly available potassium 122.7 mg kg^-1^.

### 2.2 Experiment design

Three hybrid indica rice varieties—Shenliangyou 136 (SLY136), Huiliangyousimiao (HLYSM), and Wanxiangyou982 (WXY982)—were selected as the research models. The two former varieties were widely planted in local rice production, while WXY982 presented high-yielding potential and excellent quality with ordinary lodging resistance; the three tested varieties had similar growth period. The experiment was arranged in a split plot design, with the irrigation method as the main plot and the variety as the split plot, and it was undertaken with three replicates. Rice was planted in the experimental plots surrounded by concrete ridge with 50-cm height from the soil surface, guaranteeing that the designed water layer could be achieved. The length and width of each plot were 10 and 5 m, respectively, forming a 50-m^2^ planting area; the scale of the height was marked on the inner wall of each plot in order to regulate the water depth accurately. Three irrigation modes were designed: (1) the typical high-yield irrigation scheme that is mainly based on swallow and setting (CK); (2) semi-deep water irrigation from the late tillering stage to the jointing stage (SDI1); and (3) semi-deep water irrigation from the jointing stage to the middle grain-filling stage (SDI2); meanwhile, the two semi-deep water irrigation modes are popular in the current RAAIF production, especially in rice–crayfish and rice–carp mutualistic modes. The detailed irrigation schemes of the three treatments are displayed in **Table 1**.

**Table 1:** Irrigation scheme of different rice growth periods under different irrigation treatments.

Growth period	CK	SDI1	SDI2
Recovery period	0˜2 cm WD	0˜2 cm WD	0˜2 cm WD
Tillering period	2˜4 cm WD	10˜15 cm WD	2˜4 cm WD
Drying	Drying to soil surface cracking	25˜30 cm WD	Drying to soil surface cracking
Jointing	Swallow and wet alternation	25˜30 cm WD Swallow and wet alternation	25˜30 cm WD
Heading to middle grain-filling	Swallow and wet alternation	Swallow and wet alternation	25˜30 cm WD
Middle grain-filling to maturity	Wet and dry alternation	Wet and dry alternation	Wet and dry alternation

WD, water depth.

Certified seeds were sown on 21 May, and two seedlings per hill were manually transplanted on 20 June, with a hill and row spacing of 16 cm×30 cm. For each treatment of all varieties, fertilizer N (240 kg·ha^-1^) was applied at three phases: 40% (96 kg·ha^-1^) as basal, 30% (72 kg·ha^-1^) at tillering, and 30% (72 kg·ha^-1^) at panicle initiation using urea, while 120 kg·ha^-1^P_2_O_5_ and 192 kg·ha^-1^ K_2_O were applied as the basal fertilizer using calcium superphosphate and potassium chloride, respectively. They were according to the local standard of the high-yielding procedure.

### 2.3 Plant sampling and measurement methods

#### 2.3.1 Dry matter of panicle

Ten main stems were sampled from every plot to determine the dry weight per panicle at heading and maturity, and the accumulation weight between the two stages was also calculated.

#### 2.3.2 Apparent lodging rate

The lodging area of each plot was investigated at the maturity stage. If the angle between the plant and the ground was less than 45°, it was deemed as lodging, and the lodging area as a percentage of the total area of the plot was the apparent lodging rate (ALR, %).

#### 2.3.3 Morphological traits of whole plant and basal internode

At 30 days after heading, 10 main stems were sampled from each plot to determine the fresh weight, the gravity height, and the length of each internode. The morphological characteristics of the basal culm (e.g., culm wall thickness and culm diameter) were measured at the second elongated internode from the ground. a1 and a2 represent the outer and inner diameter of the minor axis in an oval cross-section, respectively, while b1 and b2 represent those of the major axis in an oval cross-section, respectively. The culm diameter and wall thickness were calculated as follows:


Culm diameter = (b1+b2)/2

Culm wall thickness = ((a1−a2)/2 + (b1−b2)/2)/2

#### 2.3.4 Mechanics

The bending load at breaking of the basal second elongated internode was measured at a distance of 4 cm between two supporting points by using the Ookawa and Ishihara (1992) method with a pull–push dynamometer (Aikon RZ-5, Japan). Physical parameter calculations were performed according to Zhang et al. (2014) as follows:

(1) The bending moment at basal internodes by the whole plant (WP, g cm^-1^), WP=SL*×*FW, where SL is the length from the breaking basal internode to the top (cm), and FW is the fresh weight from the breaking basal internode to the top (g); (2) the breaking strength (M, g cm-1), M=F*×*L/4, where F is the force applied to break the base segment measured (kg), and L is the distance between two points (cm); (3) the cross-section modulus of the base oval hollow internode (Z, mm^3^), Z= π*/*32*×*(a1^3^b1-a2^3^b2)/a1, where a1 and a2 represent the outer and inner diameter of the minor axis in an oval cross-section, respectively, while b1 and b2 represent those of the major axis in an oval cross-section, respectively; (4) the bending stress of the material strength of culm (BS, g mm^-2^), BS=M*/*Z; and (5) lodging index LI%=WP*/*M.

#### 2.3.5 Carbohydrate content

Eight rice main stems were sampled from each plot at 30 days after heading; the NSC, lignin, and cellulose content in the culm and sheath from the basal second internode were generally determined, which was in reference to the method of Zhang et al. (2016).

#### 2.3.6 Anatomical observation of basal internode

To determine the culm anatomical structure, eight rice main stems were sampled from each plot at 30 days after heading, and the middle part of the basal second internode was excised using a razor and immediately placed in a fixing solution (70% ethanol: 5% acetic acid: 3.7% formaldehyde) for 24 h. Then, the paraffin sections were processed in accordance with the method of Du et al. (2013).

### 2.4 Statistical analysis

Microsoft Excel 2016 and Origin 10.0 (OriginLab Corp., Northampton, MA, USA) were used to produce tables and figures, respectively. Statistical analysis was conducted using SPSS 25.0. The means of treatments were compared using the least significant difference (LSD) test. The significance of irrigation management, genotype, and their interaction was measured by using Duncan’s new multiple-range test. Pearson correlation analysis was applied to investigate the relationship between lodging-resistance-related parameters.

## 3 Results

### 3.1 Dry matter accumulation of rice panicle

Variance analysis indicated that irrigation management, genotype, and their interaction all presented significant effects on the dry weight per panicle (DWPP) at heading and maturity, whereas dry weight accumulation from heading to maturity was not significantly affected by G and the interaction between G and IM (**Table 2**).

**Table 2:** Effects of semi-deep water irrigation during different periods on dry weight per panicle at heading and maturity stage.

Variety	Treatment	DW per panicle at HS (g)	DW per panicle at MS (g)	DW accumulation per panicle from HS to MS (g)
SLY136	CK	1.08a	3.55a	2.47a
	SDI1	1.00a	3.04a	2.04ab
	SDI2	0.80a	2.29b	1.50b
HLYSM	CK	1.02a	3.30a	2.28a
	SDI1	0.91a	2.70b	1.79a
	SDI2	0.76a	2.77b	2.00a
WXY982	CK	0.78a	3.30a	2.51a
	SDI1	0.57b	2.40b	1.84b
	SDI2	0.64ab	2.32b	1.69b
ANOVA				
	G	44.67**	5.86*	0.02ns
	IM	23.94**	55.68**	28.14**
	G*IM	3.22ns	4.91*	3.54ns

Different letters represent significant difference at the 0.05 level. * and **, significant at P<0.05 and P<0.01, respectively. The same as below. ns, no significant.

For SLY136, HLYSM, and WXY982 at the heading stage, the DWPP values were 7.4%, 10.8%, and 26.9% lower under SDI1 than those of CK, respectively, whereas SDI2 induced a loss of 25.9%, 24.5%, and 17.8% of DWPP, respectively, showing a stronger effect than SDI1. For SLY136, HLYSM, and WXY982 at the maturity stage, DWPP values were 21.9%, 18.0%, and 21.8% lower under SDI1 than those of CK, respectively, whereas SDI2 induced a loss of 17.4%, 18.2%, and 27.3% of DWPP, respectively, indicating that semi-deep water irrigation generally induced a larger falling range in DWPP at the heading stage than at the maturity stage. Dry weight accumulation from heading to maturity per panicle was markedly reduced by semi-deep water irrigation; SDI2 presented a stronger influence on SLY136 and WXY982 than SDI1, while SDI1 exhibited a larger effect on HLYSM than SDI2.

### 3.2 Apparent lodging rate and plant mechanics parameters

Genotype, irrigation management, and their interaction all exhibited significant effects on plant mechanics parameters.

As presented in **Table 3**, the apparent lodging rates of WXY982 were 0, 20.1%, and 95.0%, respectively, showing a significant difference between any two of the three treatments, whereas no lodging phenomenon was observed in SLY136 and HLYSM. The lodging index was a popular evaluation method for rice stem lodging resistance, and rice with a higher LI had a better lodging resistance than that with a lower LI. The three tested varieties all showed higher LIs under the two semi-deep water irrigation treatments relative to CK, reflecting that their lodging resistance was adversely influenced by semi-deep water irrigation. For SLY136, HLYSM, and WXY982, 29.2%, 26.3%, and 48.0% higher LIs were found in SDI relative to CK, while 15.4%, 10.3%, and 72.1% higher LIs were observed in SDI2 relative to CK, respectively. Breaking force values of the basal internode (F) were 29.4%, 26.1%, and 41.9% lower under SDI1 than CK for SLY136, HLYSM, and WXY982, respectively, while 22.5%, 19.4%, and 52.9% lower F values were found under SDI2 than CK for SLY136, HLYSM, and WXY982, respectively. No significant difference was observed in F between SDI1 and SDI2 for HLYSM, whereas a significant difference was detected between the two SDI treatments for SLY136 and WXY982. For all the varieties, FW showed decreasing tendency under two semi-deep water irrigation treatments when compared with CK, ranging from 9.3% to 23.3%, and the decreasing effect of SDI2 was obviously larger than that of SDI1. Meanwhile, rice grown under a semi-deep water irrigation regime showed higher SL than CK, as SDI1 increased SL by 8.1%, 3.5%, and 2.3%, respectively, while SDI2 increased SL by 16.7%, 9.8%, and 4.6%, respectively, presenting stronger impact on SL than SDI1. WP, the product of FW and SL, was decreased by both SDI1 and SDI2 for all the tested varieties, since the descending amplitude of FW was larger than the increasing amplitude of SL.

**Table 3:** Effects of semi-deep water irrigation on rice apparent lodging rate and mechanics parameters of the basal second internode.

Variety	Treatment	WP (g.cm)	FW (g)	SL (cm)	M (g·cm)	LI (%)	Breaking force (N)	ALR(%)
SLY136	CK	3575.7a	33.1a	108.0c	3198.0a	111.8c	16.0a	
	SDI1	3252.4b	27.9b	116.7b	2261.0c	144.1a	11.3c	
	SDI2	3202.4b	25.4c	126.0a	2470.5b	129.7b	12.4b	
HLYSM	CK	3309.7a	29.0a	114.0c	2675.5a	123.8c	13.4a	
	SDI1	3102.7b	26.3b	118.0b	1987.5b	156.4a	9.9b	
	SDI2	2943.5c	23.5c	125.2a	2156.0b	136.6b	10.8b	
WXY982	CK	2736.2a	21.7a	126.1c	2723.5a	100.5c	13.6a	0c
	SDI1	2345.0b	18.2b	129.0b	1577.5b	148.7b	7.9b	20.1b
	SDI2	2215.7c	16.8c	131.9a	1283.5c	173.0a	6.4c	95.0a
ANOVA								
	G	678.77**	1493.18**	179.13**	252.78**	14.08**	252.78**	
	IM	142.34**	524.51**	146.35**	456.35**	141.36**	456.35**	
	G×IM	3.25*	13.65**	13.64**	33.42**	31.74**	33.42**	

SLY136, Shenliangyou136; HLYSM, Huiliangyousimiao; WXY982, Wanxiangyou982. CK, the typical high-yield irrigation model that was mainly based on swallow and setting; SDI1, semi-deep water irrigation from late tillering to jointing; SDI2, semi-deep water irrigation from jointing to middle grain-filling; WP, bending moment by whole plant; FW, fresh weight from breaking basal internodes to top; SL, culm length from breaking basal internodes to top; M, breaking strength; IM, irrigation management; G, genotype.

Different letters represent significant difference at the 0.05 level. * and **, significant at P<0.05 and P < 0.01, respectively. The same as below.

### 3.3 Culm morphology parameters

According to variance analysis, genotype, irrigation management, and their interaction all exhibited significant influence on culm morphology parameters, besides irrigation management’ s effect on the inner diameter of the minor axis (**Table 4**).

**Table 4:** Effects of semi-deep water irrigation during different periods on rice morphology parameters of the basal second internode.

Variety	Treatment	Culm diameter(mm)	Culm wall thickness (mm)	b1(mm)	b2(mm)	a1(mm)	a2(mm)	BS (g·mm^2^)	Z(mm^3^)	Relative gravity height(%)
SLY136	CK	6.84c	0.92a	7.65c	5.26b	6.02c	3.21c	1312.2a	24.4c	43.3c
	SDI1	8.00a	0.81b	8.77a	6.34a	7.24a	4.63a	621.3c	36.5a	46.1b
	SDI2	7.60b	0.72c	8.44b	5.38b	6.78b	4.15b	761.6b	32.5b	48.0a
HLYSM	CK	7.08b	0.80a	7.89a	5.52b	6.28c	3.94b	1072.9a	25.2b	41.2c
	SDI1	7.57a	0.53b	8.07a	6.08a	7.07b	4.96b	683.2b	29.1ab	44.2b
	SDI2	8.00a	0.46b	8.46a	6.09a	7.54a	5.18a	599.1b	35.1a	46.4a
WXY982	CK	7.75b	0.92a	8.68ab	6.32a	6.82b	4.50a	874.2a	31.2b	41.7c
	SDI1	7.88b	0.66b	8.52b	5.39b	7.23a	4.04b	406.1b	38.9a	44.6b
	SDI2	8.18a	0.73b	8.94a	6.25a	7.43a	4.83a	331.2c	39.0a	46.26a
ANOVA										
	G	26.19**	40.82*	21.45**	4.08*	18.15**	20.45**	141.70**	21.95**	22.66**
	IM	63.53*	50.90**	18.67**	2.32ns	74.33**	30.56**	373.29**	42.00**	129.10**
	G×IM	13.08*	3.66*	9.18**	16.48**	8.9**	14.14**	9.77**	4.40**	0.17ns

b1 and b2 represent the outer and inner diameter of the major axis in an oval cross-section, respectively; a1 and a2 represent the outer and inner diameter of the minor axis in an oval cross-section, respectively.

Different letters represent significant difference at the 0.05 level. * and **, significant at P < 0.05 and P < 0.01, respectively. ns, no significant.

**Figure 1:**
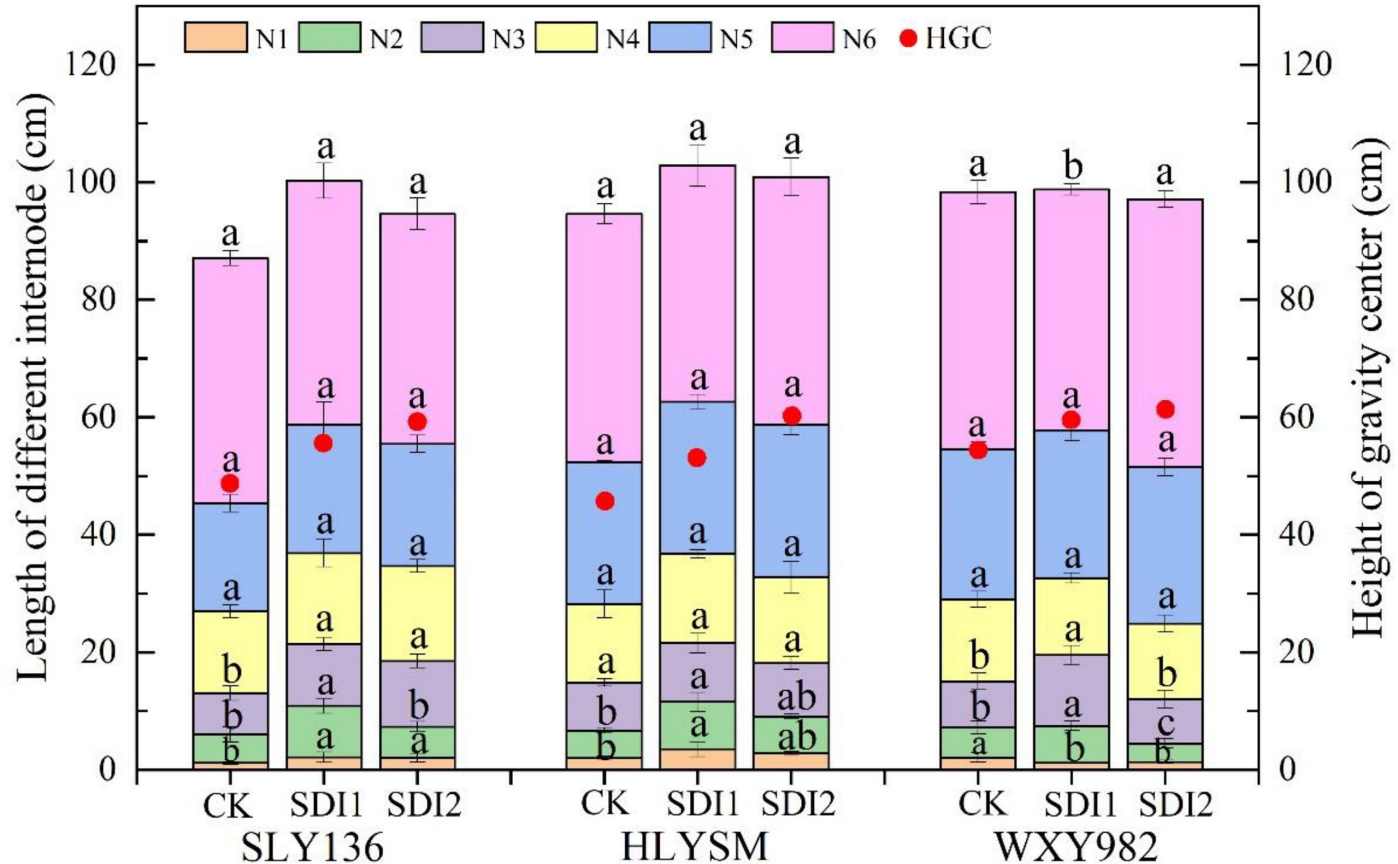
Effects of semi-deep water irrigation on configuration of rice internodes. N1, the first internode; N2, the second internode; N3, the third internode; N4, the fourth internode; N5, the fifth internode; N6, the sixth internode. Different letters represent significant difference at the 0.05 level.

As shown in **Figure 1**, the plant height of SLY136 and HLYSM was increased by the two semi-deep water irrigation treatments compared with CK, which was mainly due to the elongation of the two basal internodes; the plant height of WXY982 varied not distinctly among different irrigation treatments, but its basal third internode showed a significantly higher length under SDI1 than CK, while no obvious difference in the length of other internodes was detected across different treatments.

Morphology parameters of the basal second internode varied a lot between CK and two semi-deep water irrigation treatments (**Table 4**). When compared with CK, the Z (cross-section modulus) values of SLY136, HLYSM, and WXY982 were increased by 9.6%, 15.5%, and 24.7% under SDI1, respectively, while the Z values of SLY136, HLYSM, and WXY982 were increased by 33.2%, 39.3%, and 25.0% under SDI1, respectively. Two semi-deep water irrigation treatments induced a clear decline of BS relative to CK. The descending amplitudes caused by SDI1 were 2.6%, 36.3%, and 53.5% for SLY136, HLYSM, and WXY982, respectively, while 42.0%, 44.2%, and 62.1% descending amplitudes caused by SDI2 were detected for SLY136, HLYSM, and WXY982, respectively, showing stronger effects than SDI1. The culm diameters ordered from highest to lowest were WXY982, HLSM, and SLY136 under the same irrigation management, and they were increased by the two semi-deep water irrigation treatments relative to CK for all three varieties. Semi-deep water irrigation tended to lower the culm wall thickness, and a significant difference was detected among the three irrigation treatments for SLY136 and HLSM.

### 3.4 Contents of carbohydrates in the culm and sheath of basal internode

In accordance with variance analysis, the contents of NSC and two structural carbohydrates of the culm and sheath in the basal internode were all significantly affected by genotype, irrigation management, and their interaction, except that NSC in sheath was not significantly affected by genotype (**Table 5**).

**Table 5:** Effects of semi-deep water irrigation during different periods on structural carbohydrate content of rice basal second internode.

Variety	Treatment	Culm			Sheath		
		NSC (%)	Cellulose (%)	Lignin (%)	NSC (%)	Cellulose (%)	Lignin (%)
SLY136	CK	16.1a	33.2a	17.9a	11.9a	35.6a	18.5a
	SDI1	12.9b	31.0ab	16.6b	9.4b	32.7b	17.3a
	SDI2	10.2c	27.6b	15.3c	7.1c	30.0c	17.5a
HLYSM	CK	28.0a	27.7a	16.2a	20.5a	35.8a	14.8a
	SDI1	22.0b	26.1a	15.6a	14.7b	33.4b	13.4a
	SDI2	24.0b	25.4a	13.9a	11.1c	29.7c	13.8a
WXY982	CK	20.4a	21.3a	8.5a	16.9a	23.9a	7.4a
	SDI1	20.0a	20.6a	7.5b	7.1c	22.7a	6.1b
	SDI2	15.7b	17.5b	6.3c	10.4b	18.9b	5.2c
ANOVA							
	G	23.95**	294.34**	12.75**	2.36ns	466.23**	61.31**
	IM	47.64**	187.94**	183.28**	82.92**	448.59**	327.23**
	G×IM	65.27**	54.57**	95.29**	27.52**	174.37**	163.28**

Different letters represent significant difference at the 0.05 level. **, significant at P < 0.01. ns, no significant.

Two semi-deep water irrigation treatments obviously lowered the NSC content besides WXY982 under SDI1 compared with CK. For SLY136, HLYSM, and WXY982, the descending amplitudes of the lignin content in culm under SDI1 were 6.6%, 5.8%, and 3.3%, respectively, while larger descending amplitudes were found in the effect of SDI2, which were 16.9%, 8.3%, and 17.8%, respectively. Similar with culm, the sheath lignin content was reduced more clearly by SDI2 than SDI1. Generally, the cellulose content of culm and sheath was reduced by both SDI1 and SDI2 compared with CK, and we found a larger falling percentage in culm than in sheath. Under the same irrigation management, WXY982 had lower lignin and cellulose content in culm and sheath than the other two varieties, showing worse structural carbohydrate accumulation ability.

### 3.5 Anatomy structure of basal internode

**Figure 2:**
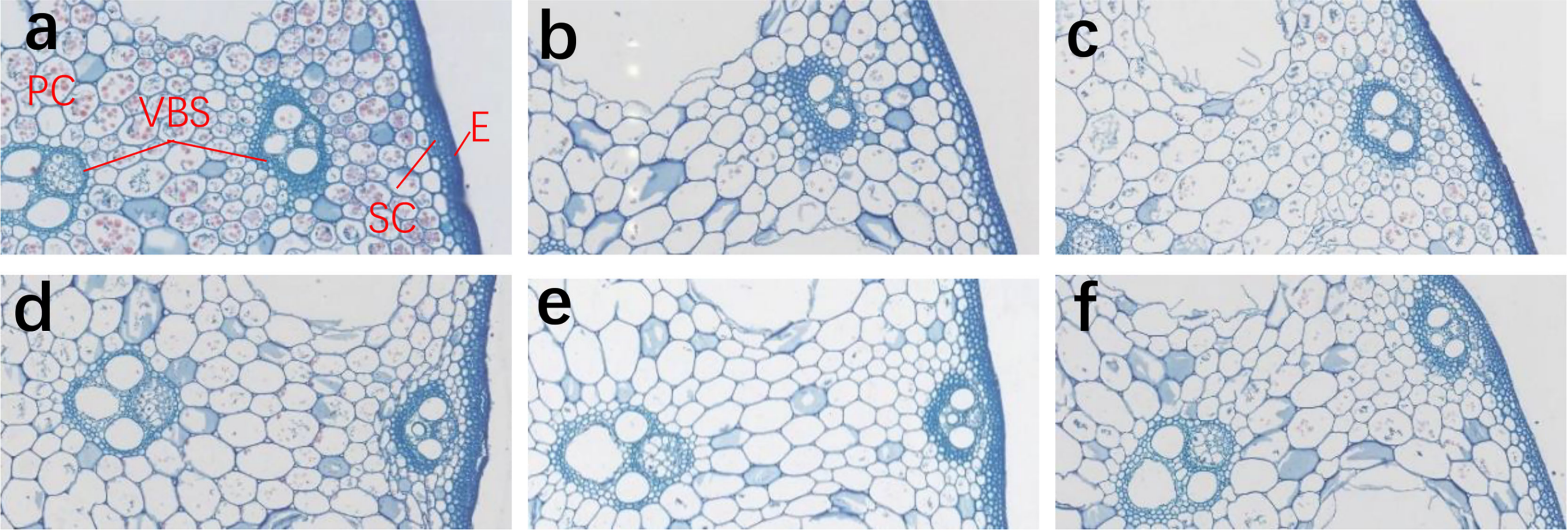
The cross-section of the basal second internode of HLYSM and WXY982 under different irrigation treatments. The figure showed the hundred-fold imagination of the basal internode cross-section under microscope. **(A–C)** represent CK, SDI1, and SDI2 of HLYSM, respectively; **(D–F)** represent CK, SDI1, and SDI2 of WXY982, respectively. PC, parenchymal cell; SC, sclerenchyma cell; VBS, vascular bundle sheath; AC, air cavity; MC, medullary cavity.

**Figure 2** shows the representative cross-section imagination of the basal internode for HLYSM and WXY982 under three irrigation treatments, and **Table 6** presents the detailed related data. Aside from the fact that genotype had no significant effects on the number of small vascular bundles, genotype, irrigation management, and their interaction all exhibited significant influence on each parameter. Compared with CK, two semi-deep water irrigation treatments significantly enlarged the stomata area for both rice varieties, and the number of stomata was significantly reduced by SDI1 and SDI2 for HLYSM and WXY982, respectively. Parenchymal cells tended to have a larger area and were arranged more loosely under two semi-deep water irrigation treatments than CK. Compared with CK, the number and area of both large and small vascular bundles generally showed a decreasing tendency under SDI1 and SDI2. Moreover, the basal internode grown under the two semi-deep water irrigation regimes presented a lower degree of densification and a less filled substance than CK. We also compared the difference of the anatomy characteristics between the two tested varieties and found that HLYSM had more compact sclerenchyma and its parenchymal cells were arranged more closely than WXY982; moreover, a larger area of large and small vascular bundles in HLYSM than in WXY982 was detected.

**Table 6:** Effects of semi-deep water irrigation during different periods on vascular bundle characteristics and air cavity of rice basal second internode.

Variety	Treatment	Number of big vascular bundles	Area of big vascular bundles (mm^2^)	Number of small vascular bundles	Area of small vascular bundles(mm^2^)	Stoma area(mm^2^)	Number of stoma	Number of cell per mm^2^
HLYSM	CK	35.6a	0.142a	32.7a	0.089a	0.376c	31.0a	162.3a
	SDI1	31.3c	0.102b	29.7b	0.075c	0.990a	27.0b	129.4b
	SDI2	33.3b	0.139a	31.0a	0.081b	0.463b	30.3a	130.5b
WXY982	CK	33.3a	0.107a	31.7a	0.067a	0.345c	30.7a	138.6a
	SDI1	32.0a	0.078c	30.7ab	0.034b	0.511b	30.3a	86.9b
	SDI2	28.3b	0.091b	29.0bc	0.063a	0.738a	28.0b	84.3c
ANOVA								
	G	44.44**	87.46**	1.29ns	772.74**	57.22**	0.08ns	1013.12**
	IM	44.33**	26.52**	28.43**	215.79**	480.23**	7.00*	583.79**
	G*IM	24.11**	5.39*	16.71**	48.59**	447.5**	25.92**	34.89**

Different letters represent significant difference at the 0.05 level. * and **, significant at P < 0.05 and P < 0.01, respectively. ns, no significant.

### 3.6 Correlation analysis

Correlation analysis showed that the LI had a significant negative relationship with M for the three varieties, and the correlation coefficient exceeded 0.9 (**Table 7**). A significant positive relationship was detected between M and BS. For all varieties, M was generally found to correlate significantly positively with the plant height, gravity height, culm diameter, and Z. There was a significant positive relationship between M and culm wall thickness, basal internode lignin, and cellulose content, while the lignin content of the basal internode culm and the cellulose content of the basal internode sheath presented no significant relationship with M for HLYSM.

**Table 7:** Correlation analysis of lodging-resistant parameters with lodging index and breaking strength of basal second internode.

Parameters	SLY136		HLYSM		WXY982	
	LI	M	LI	M	LI	M
M	-0.958**	1	-0.908**	1.000	-0.976**	1.000
LI	1.000	-0.958**	1.000	-0.908**	1.000	-0.976**
Breaking force of basal second internode	-0.958**	1.000**	-0.908**	1.000**	-0.976**	1.000**
Breaking force of basal third internode	-0.716**	0.861**	-0.687*	0.861**	-0.944**	0.966**
Plant height	0.684*	-0.858**	0.359	-0.664*	0.625*	-0.685*
Gravity height	0.639*	-0.816**	0.386	-0.684*	0.944**	-0.967**
Culm wall thickness	-0.501	0.673*	-0.540	0.812**	-0.607*	0.722**
Culm diameter	0.924**	-0.963**	0.307	-0.597*	0.801**	-0.762**
Length of basal second internode	0.636*	-0.590*	0.817**	-0.791**	-0.339	0.276
Length of basal third internode	0.669*	-0.781**	0.439	-0.499	0.103	-0.219
Z	0.813**	-0.884**	0.140	-0.463	0.789**	-0.865**
BS	-0.910**	0.980**	-0.637*	0.861**	-0.953**	0.993**
Culm cellulose of basal second internode	0.925**	-0.948**	-0.474	0.726**	-0.875**	0.807**
Culm lignin of basal second internode	0.293	-0.134	-0.097	0.425	-0.957**	0.930**
Sheath cellulose of basal second internode	0.937**	-0.970**	-0.681*	0.805**	-0.839**	0.792**
Sheath lignin of basal second internode	0.379	-0.447	-0.615*	0.749**	-0.944**	0.923**

* and **, significant at P < 0.05 and P < 0.01, respectively.

## 4 Discussion

### 4.1 Influence of semi-deep water irrigation during different growth periods on rice lodging resistance and genotype difference

The scale of rice-aquatic animal integration increased rapidly, which gained a favorable and friendly relationship with the environment and had developed to a major rice cropping pattern in China; semi-deep water irrigation was carried out to meet the requirement of aquatic animal culture for a given rice growth period under RAAIF. Rice cultivated under RAAIF showed higher lodging frequency than rice monoculture, and nearly all belonged to stem lodging based on our survey. Therefore, we compared the rice stem lodging resistance performance between three irrigation managements: the treatment of CK based on swallow, which represents a high-yielding irrigation scheme, and two representative semi-deep water irrigation modes—one occurs from the rice tillering stage to the booting stage, and another occurs from the jointing stage to the middle grain-filling stage.

The lodging index is an evaluation methodology that correlates the mechanics and morphology of the rice stem, which was widely used in the analysis of rice stem lodging resistance. The lodging index is a specific value of WP to M, and WP is the product of SL and FW. Compared with traditional irrigation based on swallow and wetting, semi-deep water irrigation during different growth periods reduced WP and M and increased LI, resulting in weaker lodging resistance. Results suggested that semi-deep water irrigation inhibited the translocation from the vegetative organ to the panicle, thereby causing yield loss (**Supplementary Table 1**). In this experiment, leaves located at the lower part of the rice plant were submerged under semi-deep water irrigation, and we observed higher-position tillers occurring frequently. The higher-position tillers would consume more nutrients while forming panicles with little spikelets and would fail to reach maturity, actually causing a negative effect on dry matter accumulation and the grain filling of mother culms. Meanwhile, semi-deep water irrigation prolonged the length of the basal internode so as to increase SL, which was consistent with the studies that also involved the response of the rice internode length to deep-water irrigation scenarios (Anandan et al., 2012; Zhu et al, 2019). Previous studies have certified that rice internode elongation was related with its higher gibberellin content that was mediated by more ethylene release, which was induced by oxygen deficit under waterlogging (Sauter and Kende, 1992; Hattori et al., 2009; Singh et al., 2009). In this research, the declining amplitude of FW exceeded the increasing amplitude of SL under semi-deep water irrigation relative to CK, inducing a decreased WP. M was actually determined through the breaking force of the basal internode (F). In this study, F was decreased by semi-deep water irrigation, and its declining amplitude exceeded WP markedly, leading to the increased LI. Among the three tested varieties, the F value of WXY982 declined more drastically under semi-deep water irrigation, resulting in a higher descending amplitude in M and an increasing amplitude in LI compared with HLYSM and SLY136. Although the culm of the three varieties exhibited a similar mechanics response to semi-deep water irrigation, SLY136 and HLYSM were affected more deeply by SDI1, while WXY showed a more sensitive response to SDI2, which was mainly linked to the variance between the breaking force of the basal internode.

### 4.2 The physical mechanism for weaker stem mechanical strength exposed to semi-deep water irrigation

Increasing the mechanical strength of the culm of the basal internodes is important to reduce rice lodging risks (Shah et al., 2019), especially for indica rice varieties with high aboveground biomass. The breaking force of the basal internode was generally used to reflect the rice mechanical strength. It is quite crucial for the mechanical strength enhancement of crop culm by increasing the thickness of the mechanical tissue, area, and compactness of vascular bundles and improving the lignification degree of vascular bundle sheaths (Wang et al., 2015; Zhang et al., 2016; Zheng et al., 2017). Based on the anatomical observation in this study, semi-deep water irrigation reduced the thickness of mechanical tissues, sclerenchyma cells, and parenchymal cells; reduced the number of vascular bundles; and caused a looser arrangement, thereby weakening the fullness of the rice basal internode. The rice plant body is principally supported by structural carbohydrate, especially lignin and cellulose, and their accumulation level is considered as a key factor in culm mechanical strength determination (Kashiwagi et al., 2008; Zhang et al., 2010 Rice science; Zhang et al., 2014). Our study indicated that semi-deep water irrigation lowered the lignin and cellulose content in the culm and sheath of the rice basal internode for all varieties, and they were positively correlated with the breaking force and negatively correlated with the lodging index, as proved by correlation analysis. Thus, we thought that the decline in the contents of the two structural carbohydrate components probably contributed to the weaker mechanical strength of culms. For future research, rice culm lignin and cellulose synthesis pathway under semi-deep water irrigation should be investigated deeply. We had also observed anatomy difference between varieties. The recognized lodging-resistant variety HLYSM presented more numbers and an area of vascular bundles, more compact cell arrangement, and filled substance of the basal internode when compared with WXY982, which facilitates better development of mechanical tissues. Generally, the anatomy performance of the basal internode varied a little between semi-deep water irrigation during different rice growth periods for a given rice variety.

The mechanical tissue in the basal internode of both HLYSM and WXY982 tended to develop more weakly under two semi-deep water irrigation treatments. Marked lodging occurred in WXY982, while no lodging occurrence was detected in HLYSM, and it may be linked to its better fundamental mechanical tissue development than WXY982. Three basal internodes prolonged approximately across 15 days after jointing; thereby both SDI1 and SDI2 covered the filling periods of two or three basal internodes in this study. Actually, the substance for rice basal internode filling derives from three adjacent leaves, namely one upper leaf, one lower leaf, and one enfolding the internode, and leaves located at the lower part of the rice plant could only receive much less illumination under semi-deep water irrigation since they were soaked in water, which would suppress glucose synthesis and vitamin B, substrates of lignin and cellulose synthesis, due to their poor light interception (Taylor, 2008). Consequently, we speculated that the declined lignin and cellulose accumulation of the basal internode under semi-deep irrigation was mainly attributed to the light deficiency of soaked basal leaves. Other studies also certified that the weak filling degree of basal internodes in crops was closely related to insufficient lignin when the lower part of the plant received less radiation (Xue et al., 2016; Wu et al., 2017; Hussain et al., 2019). Results of correlation analysis proved the negative relationship between structural carbohydrates and breaking force in the rice basal internode as well. Besides stem, root characteristics ought to be explored in the future since its anchorage also played an important role in rice lodging resistance determination, and its sensitivity to water regime had been reported in previous literature (Kato et al., 2006; Mishra and Salokhe, 2010; Nishiuchi et al., 2012). To cope with the inevitable weaker rice lodging resistance under semi-deep water irrigation, it is necessary to select the rice variety with favorable lodging resistance and deal with appropriate integrated farming phase and cultivation measures in consideration of the requirement for aquatic animal culture and ensuring of adequate rice lodging resistance under RAAIF.

## 5 Conclusion

Semi-deep water irrigation decreased the WP and M of the rice stem and induced the increased lodging index due to the larger amplitude of variation in M than WP under both SDI1 and SDI2. The degree of influence of semi-deep water irrigation on the lodging index during different phases depended on the specific rice variety. The worse mechanical strength of the rice basal internode under semi-deep water irrigation was closely connected with weaker vascular bundle development and suppressed structural carbohydrate accumulation.

## Data availability statement

The raw data supporting the conclusions of this article will be made available by the authors, without undue reservation.

## Author contributions

HG and ZD designed the experiment. ZD, LC, and YL performed the research. DS, RS, and XC assisted in experiments or data analysis. HG, ZD, LC, and QX wrote the draft manuscript. All authors contributed to the article and approved the submitted version.

## References

[B1] Anandan A. Rajiv G. Ramarao A. Prakash M. (2012). Internode elongation pattern and differential response of rice genotypes to varying levels of flood water. Funct. Plant Biol. 39, 137–145. doi: 10.1071/FP11184 32480768

[B2] Belder P. Spiertz J. H. J. Bouman B. A. M. Lu G. Tuong T. P. (2005). Nitrogen economy and water productivity of lowland rice under water-saving irrigation. Field Crops Res. 93, 169–185. doi: 10.1016/j.fcr.2004.09.022

[B3] Chu M. Liu M. Ding Y. Wang S. Liu Z. Tang S.. (2017). Effect of nitrogen and silicon on rice submerged at tillering stage. Agron. J. 110, 183–192. doi: 10.2134/agronj2017.03.0156

[B4] Du H. F. Zheng Z. H. Zhao H. F. (2013). Method of paraffin section of garlic scape in its maturity and the anatomical structure. Res. Explor. Lab. 32, 17–20.

[B5] Frei M. Becker K. (2005). Integrated rice-fish culture: Coupled production saves resources. Nat. Resour. Forum. 29, 135–143. doi: 10.1111/j.1477-8947.2005.00122.x

[B6] Gui M. Wang D. Xiao H. Tu M. LI F. Li W.. (2018). Studies of the relationship between rice stem composition and lodging resistance. J. Agr. Sci. 156, 387–395. doi: 10.1017/S0021859618000369

[B7] Hattori Y. Nagai K. Furukawa S. Song X. J. Kawano R. Sakakibara H.. (2009). The ethylene response factors *SNORKEL1* and *SNORKEL2* allow rice to adapt to deep water. Nature. 460, 1026–1030. doi: 10.1038/nature08258 19693083

[B8] Hou J. Styles D. Cao Y. Ye X. (2021). The sustainability of rice-crayfish coculture systems: a mini review of evidence from jianghan plain in China. J. Sci. Food Agr. 101, 3843–3853. doi: 10.1002/jsfa.11019 33336495

[B9] Hussain S. Iqbal N. Ting P. Khan M. N. Liu W. Yang W. (2019). Weak stem under shade reveals the lignin reduction behavior. J. Integr. Agr. 18, 496–505. doi: 10.1016/S2095-3119(18)62111-2

[B10] Kashiwagi T. Togawa E. Hirotsu N. Ishimaru K. (2008). Improvement of lodging resistance with QTLs for stem diameter in rice (Oryza sativa l.). Theor. Appl. Genet. 117 (5), 749–757. doi: 10.1007/s00122-008-0816-1 18575836

[B11] Kato Y. Abe J. Kamoshita A. Yamagishi J. (2006). Genotypic variation in root growth angle in rice (Oryza sativa l.) and its association with deep root development in upland fields with different water regimes. Plant Soil. 287 (1), 117–129. doi: 10.1007/s11104-006-9008-4

[B12] Kende H. Knaap E. V. D. Cho H. T. (1998). Deepwater rice: a model plant to study stem elongation. Plant Physiol. 118 (4), 1105–1110. doi: 10.1104/pp.118.4.1105 9847084 PMC1539197

[B13] Liu Q. Ma J. Zhao Q. Zhou X. (2018). Physical traits related to rice lodging resistance under different simplified-cultivation methods. Agron. J. 110, 127–132. doi: 10.2134/agronj2017.09.0548

[B14] Liu J. Wang Q. Yuan J. Zhang T. Ye S. Li W.. (2018). Integrated rice-field aquaculture in China, a long-standing practice, with recent leapfrog developments. Aquaculture China: Success stories modern trends. 2018, 174–184. doi: 10.1002/9781119120759.ch2_6

[B15] Mishra A. Salokhe V. M. (2010). Flooding stress: The effects of planting pattern and water regime on root morphology, physiology and grain yield of rice. J. Agron. Crop Sci. 196, 368–378. doi: 10.1111/j.1439-037X.2010.00421.x

[B16] Mulsanti I. W. Yamamoto T. Ueda T. Samadi A. F. Kamahora E. Rumanti I. A.. (2018). Finding the superior allele of japonica-type for increasing stem lodging resistance in indica rice varieties using chromosome segment substitution lines. Rice. 11, 1–14. doi: 10.1186/s12284-018-0216-3 29671092 PMC5906422

[B17] National Fisheries Technology Extension Center (2021). Report on rice-fish integrated farming industry development during “thirteen five” in China. (Beijing: China Fisheries).

[B18] Nishiuchi S. Yamauchi T. Takahashi H. Kotula l. Nakazono M. (2012). Mechanisms for coping with submergence and waterlogging in rice. Rice. 5, 1–14. doi: 10.1186/1939-8433-5-2 24764502 PMC3834488

[B19] Ohe M. Okita N. Daimon H. (2010). Effects of deep-flooding irrigation on growth, canopy structure and panicle weight yield under different planting patterns in rice. Plant Prod. Sci. 13, 193–198. doi: 10.1626/pps.13.193

[B20] Ookawa T. Ishihara K. (1992). Varietal difference of physical characteristics of the culm related to lodging resistance in paddy rice. Japanese J. Crop Science. 61 (3), 419–425. doi: 10.1626/jcs.61.419

[B21] Ookawa T. Inoue K. Matsuoka M. Ebitani T. Takarada T. Yamamoto T.. (2014). Increased lodging resistance in long-culm, low-lignin gh2 rice for improved feed bioenergy production. Sci. Rep-UK. 4, 1–9. doi: 10.1038/srep06567 PMC4190510 25298209

[B22] Sauter M. Kende H. (1992). Gibberellin-induced growth and regulation of the cell division cycle in deepwater rice. Planta. 188 (3), 362–368. doi: 10.1007/BF00192803 24178326

[B23] Shah L. Yahya M. Shah S. M. A. Nadeem M. Ali A. Ali A.. (2019). Improving lodging resistance: Using wheat and rice as classical examples. Int. J. Mol. Sci. 20, 4211. doi: 10.3390/ijms20174211 31466256 PMC6747267

[B24] Singh S. Mackill D. J. Ismail A. M. (2009). Responses of *SUB1* rice introgression lines to submergence in the field: yield and grain quality. Field Crops Res. 113 (1), 12–23. doi: 10.1016/j.fcr.2009.04.003

[B25] Taylor N. G. (2008). Cellulose biosynthesis and deposition in higher plants. New Phytol. 178 (2), 239–252. doi: 10.1111/j.1469-8137.2008.02385.x 18298430

[B26] Wang C. Ruan R. Yuan X. Hu D. Yang H. Li Y.. (2015). Effects of nitrogen fertilizer and planting density on the lignin synthesis in the culm in relation to lodging resistance of buckwheat. Plant Prod. Sci. 18 (2), 218–227. doi: 10.1626/pps.18.218

[B27] Wang H. Zhang Y. Zhang Y. McDaniel M. D. Sun L. Su W.. (2020). Water-saving irrigation is a ‘win-win’management strategy in rice paddies–with both reduced greenhouse gas emissions and enhanced water use efficiency. Agr. Water Manage. 228, 105889. doi: 10.1016/j.agwat.2019.105889

[B28] Wu L. Zhang W. Ding Y. Zhang J. Cambula E. D. Weng F.. (2017). Shading contributes to the reduction of stem mechanical strength by decreasing cell wall synthesis in japonica rice (Oryza sativa l.). Front. Plant Sci. 8. doi: 10.3389/fpls.2017.00881 PMC5447739 28611803

[B29] Xie J. Hu L. Tang J. Wu X. Li N. Yuan Y.. (2011). Ecological mechanisms underlying the sustainability of the agricultural heritage rice–fish coculture system. P. Natl. Acad. Sci. U.S.A. 108 (50), 1381–1387. doi: 10.1073/pnas.1111043108 PMC3250190 22084110

[B30] Xue J. Gou L. Zhao Y. Yao M. Yao H. Tian J.. (2016). Effects of light intensity within the canopy on maize lodging. Field Crops Res. 188, 133–141. doi: 10.1016/j.fcr.2016.01.003

[B31] Yao F. Huang J. Cui K. Nie L. Xiang J. Liu X.. (2012). Agronomic performance of high-yielding rice variety grown under alternate wetting and drying irrigation. Field Crops Res. 126, 16–22. doi: 10.1016/j.fcr.2011.09.018

[B32] Ye Y. Liang X. Xin X. Chen Y. Liu J. Gu J.. (2013). Alternate wetting and drying irrigation and controlled-release nitrogen fertilizer in late-season rice. effects on dry matter accumulation, yield, water and nitrogen use. Field Crops Res. 144, 212–224. doi: 10.1016/j.fcr.2012.12.003

[B33] Zhang F. Jin Z. Ma J. Shang W. Liu H. Xu M.. (2010). Relationship between lodging resistance and chemical contents in culms and sheaths of japonica rice during grain filling. Rice Sci. 17, 311–318. doi: 10.1016/S1672-6308(09)60032-9

[B34] Zhang J. Li G. Song Y. Liu Z. Tang S. Zheng C.. (2014). Lodging resistance characteristics of high-yielding rice populations. Field Crops Res. 161, 64–74. doi: 10.1016/j.fcr.2014.01.012

[B35] Zhang W. Wu L. Wu X. Ding Y. Li G. Li J.. (2016). Lodging resistance of japonica rice (Oryza sativa l.): morphological and anatomical traits due to top-dressing nitrogen application rates. Rice. 9, 31. doi: 10.1186/s12284-016-0103-8 27369289 PMC4930438

[B36] Zheng M. Chen J. Shi Y. Li Y. Yin Y. Yang D.. (2017). Manipulation of lignin metabolism by plant densities and its relationship with lodging resistance in wheat. Sci. Rep-UK. 7, 1–12. doi: 10.1038/srep41805 PMC5288770 28150816

[B37] Zhu G. Chen Y. Ella E. S. Ismail A. M. (2019). Mechanisms associated with tiller suppression under stagnant flooding in rice. J. Agron. Crop Sci. 205, 235–247. doi: 10.1111/jac.12316

