# Supplementary material for: Effects of semi-deep water irrigation on hybrid indica rice lodging resistance
Source: Front Plant Sci. 2022 Dec 15;13:1038129. doi: 10.3389/fpls.2022.1038129 (PMC9798435; doi:10.3389/fpls.2022.1038129)
Supplement: Supplementary file 1 [file DataSheet_1.docx]

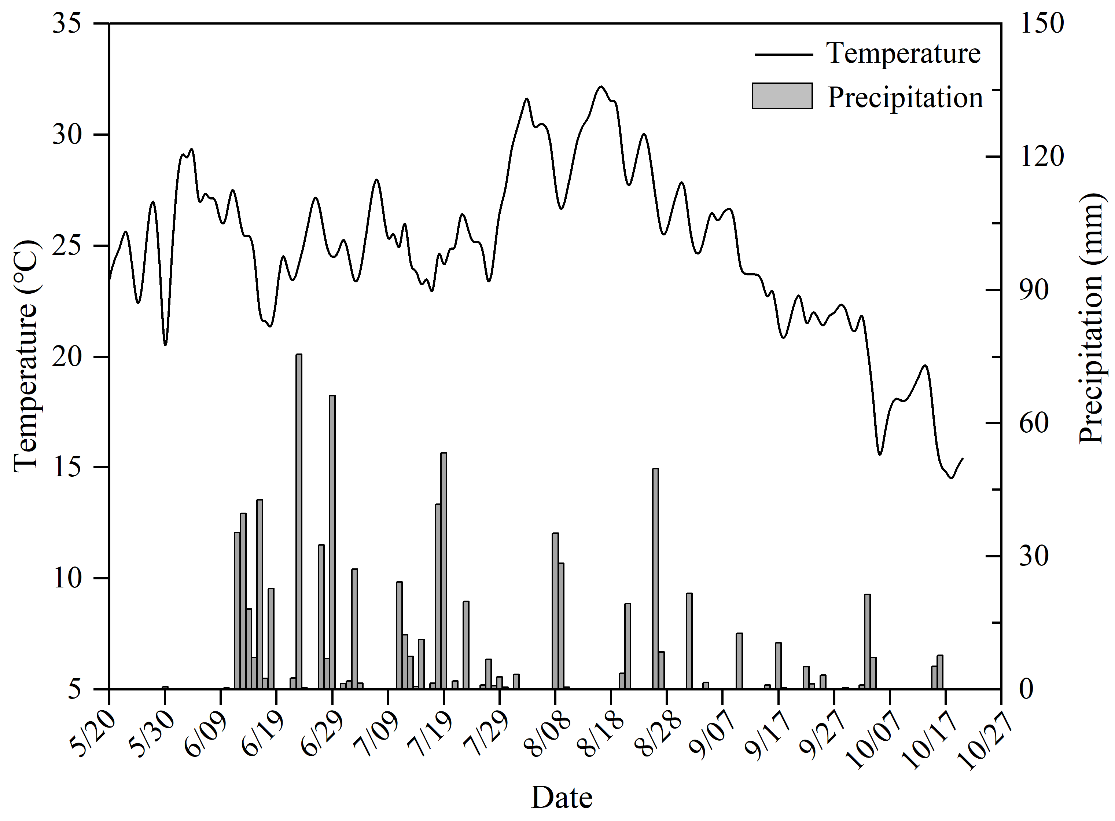


Additional Figure 1 Temperature and precipitation during rice growth period

Additional Table 1 Effects of semi-deep water irrigation during different periods on rice yield and its components

| Variery | Treatment | Panicles | Spikelets per panicle | Filled grain rate (%) | 1000-grain weight  (g) | Theoretical yield  (t•hm^-2^) | Harvest yield  (t•hm^-2^) |
| --- | --- | --- | --- | --- | --- | --- | --- |
| SLY136 | CK | 296.7a | 212.0a | 87.2a | 21.6a | 11.9a | 10.2a |
|  | SDI1 | 295.9a | 197.0b | 83.3b | 21.3a | 10.3b | 9.4ab |
|  | SDI2 | 297.8a | 198.4b | 79.8c | 20.5a | 9.7b | 9.3b |
| HLYSM | CK | 292.9a | 232.6a | 83.9a | 20.4a | 11.7a | 11.4a |
|  | SDI1 | 277.7b | 205.9b | 82.6ab | 20.9a | 9.9b | 9.8b |
|  | SDI2 | 286.1ab | 203.3b | 80.4b | 20.5a | 9.6b | 9.2b |
| WXY982 | CK | 304.2b | 192.1a | 85.5a | 21.4a | 10.7a | 10.4a |
|  | SDI1 | 313.0ab | 177.6b | 83.9a | 22.1a | 10.3a | 9.5ab |
|  | SDI2 | 315.1a | 174.2b | 80.8b | 20.7a | 9.2b | 9.0b |

Different letters represent significant difference at the 0.05 level. * and **, significant at P<0.05 and P<0.01, respectively. The same as below.
